# Feasibility of diffuse Raman spectroscopy to detect in-vivo molecular changes in the tissue induced by subcutaneous implants

**DOI:** 10.1364/BOE.567960

**Published:** 2025-08-22

**Authors:** Max Dooley, Jeni Luckett, Nga Tsing Tang, Morgan R. Alexander, Pavel Matousek, Hamid Dehghani, Yves Bayon, Amir M. Ghaemmaghami, Ioan Notingher

**Affiliations:** 1 School of Physics and Astronomy, University of Nottingham, University Park, Nottingham NG7 2RD, UK; 2 School of Life Sciences, University of Nottingham, University Park, Nottingham NG7 2RD, UK; 3 School of Pharmacy, University of Nottingham, University Park, Nottingham NG7 2RD, UK; 4 Central Laser Facility, Research Complex at Harwell, STFC Rutherford Appleton Laboratory, UK Research and Innovation (UKRI), Harwell Oxford OX11 0QX, UK; 5 School of Computer Science, University of Birmingham, Birmingham B15 2TT, UK; 6 Sofradim Production – 116 Avenue du Formans – 01600 Trévoux, France

## Abstract

Biomaterials are being designed for a broad range of medical applications, but regardless of their endpoint use, they trigger host immune responses, also known as foreign body responses (FBR). This study investigates the feasibility of using diffuse Raman spectroscopy (DRS) to detect *in-vivo* molecular changes in subcutaneous tissue situated immediately below the dermis. A mouse model of FBR after subcutaneous implantation of woven polyethylene terephthalate (PET) fibres is used to investigate this approach. Numerical modelling of light propagation in tissue is used to optimise the DRS instrument to detect Raman signals related to FBR-induced collagen deposition in tissue surrounding the PET fibres. Post-mortem *in-situ* measurements on mice (day 14, 21 and 28 post-implantation) showed that the intensity of the 930 cm^−1^ Raman band, corresponding to collagen, correlates with the level of FBR observed by histology. The optimised DRS instrument allows the acquisition of *in-vivo* spectra from on mice under anaesthesia (day 7 and 28 post-implantation). Spectra from live mice (*in-vivo*) show a similar correlation between the intensity of the 930 cm^−1^ collagen Raman band with the level of FBR, in agreement with the histology assessment of the skin. These results demonstrate the feasibility of performing *in-vivo* DRS measurements on a mouse model to detect molecular changes caused by FBR in the subcutaneous region below the skin. Such measurements can help the design of new biomaterials since it would enable longitudinal measurements of FBR on an individual animal rather the sacrificing animals for histology at specific time endpoints. Furthermore, it is amenable to development for monitoring FBR of implants clinically.

## 1. Introduction

A key challenge in developing new biomaterials for medical applications is to adequately control the Foreign Body response (FBR) to support the intended therapeutic actions of the biomaterials. FBR is an unavoidable tissue response triggered by biomaterials implanted into the body. After the implantation of biomaterials, a FBR is typically initiated, which includes the formation of a fibrotic capsule around the implant. This capsule is part of the body’s attempt to isolate the foreign object and is not inherently pathological. In many cases, a thin, loosely organized capsule is considered a normal and manageable outcome. However, when the fibrotic reaction becomes excessive, it can lead to the formation of a thick, dense, and contractile capsule that completely surrounds the implant. This abnormal response—often referred to as capsular fibrosis—can mechanically isolate the implant, interfere with its intended function, and even lead to clinical complications such as pain, deformation, or device failure [1]. The most standard method for evaluating FBR in animal models is histological assessment of *ex-vivo* subcutaneous tissue surrounding the implant. The structural organization of fibrotic capsule and infiltration of immune cells in the tissue surrounding the implant (inflammation) are two key histological parameters. Even though histology is an extremely powerful and valuable technique, its main drawback is that it is an endpoint technique that requires irreversible excision of tissue. While several imaging modalities can be used for *in-vivo* imaging in small and large animals (e.g. X-ray computed tomography, magnetic resonance imaging, ultrasound, optical coherence tomography, photoacoustic imaging), they generally lack the chemical specificity and sensitivity required to monitor biomolecular changes in tissue related to FBR.

Raman spectroscopy has been widely used for *in-vivo* measurements of skin, including animals and humans [2]. In *ex-vivo* measurements of tissue, Raman spectroscopy was able to detect and image infiltration of immune cells during inflammation of dermis, and changes in collagen structure [3]. In conventional Raman micro-spectroscopy, the information is recorded predominantly from the surface of the sample by detecting primarily the ballistic photons - photons reaching the detector after travelling in straight-line paths. This approach provides the maximum spatial resolution and spectral contrast (i.e. signal from the region of interest relative to background signal from surrounding parts of the sample). However, when attempting to detect molecular changes deeper under the surface of an optically turbid sample (e.g. sample with high level of optical scattering, such as skin), both the spatial resolution and spectral contrast rapidly degrade with increasing depth. This occurs because the scattered photons will consist of both ballistic and diffuse photons (photons that underwent multiple scattering events). Therefore, *in-vivo* confocal Raman microscopy is limited to the outmost 100 µm layer of skin [4] and is unsuitable for probing much deeper into tissue. Although using an axicon to form Bessel beams has allowed measurements of depth-resolved Raman spectra of rat skin *ex-vivo* it still limited to 100-200 µm depth [5].

The diffuse nature of photon migration in connective tissue for near-infrared light (785-1000 nm wavelength) makes diffuse Raman spectroscopy (DRS) ideal for probing deeper in skin and subcutaneous tissue (tens of millimetres), to measure the biomolecular processes during FBR. Spatially Offset Raman Spectroscopy (SORS) [6] and similar diffuse Raman approaches have been demonstrated for monitoring and quantifying collagen in bone [7] and 3D cartilage grafts grown *in-vitro* [8]. SORS based on spatial light modulators (SLMs) have provided flexibility to optimise the configuration of the laser excitation and Raman detection points on the sample surface [9], and was used to quantify the density of collagen in samples implanted under the skin of rat cadavers, mimicking collagen deposition during healing of bone defects [10]. SORS combined with partial least square (PLS) models successfully retrieved the overall pattern of collagen concentration across the concentration range simulating all stages of bone healing [11].

More recently, numerical modelling of light propagation in tissue was used to optimise and improve spectral contrast and signal-to-noise ratio (SNR) when investigating regions of interest located 0-4.5 mm below the surface of solid turbid samples [12]. The models were applied to develop a DRS instrument with increased SNR while ensuring safe laser exposure parameters required for *in-vivo* measurements by calculating the maximum permissible exposure (MPE). Using phantom samples (polymer disks coated with collagen) implanted post-mortem in mice to mimic the formation of the fibrotic capsule in typical FBR, the instrument achieved a limit of detection of ∼20 μm for the thickness of fibrotic layer [11].

This paper investigates the feasibility of using DRS for *in-vivo* measurements on mice to detect biomolecular changes in skin as induced by FBR after subcutaneous implantation of biomaterials. After further optimisation of the instrument to increase optical, DRS measurements were carried out at different time-points after implantation of polyethylene terephthalate (PET) woven fibres. The information extracted from the DRS data was compared to reference histology and collagen tissue staining to correlate the observed changes in the Raman spectra with the molecular changes in tissue induced by FBR, to demonstrate the applicability of this technique for continuous non-invasive tissue response.

## 2. Material and methods

### 2.1. Animal models and samples

All animal experiments were approved following local ethical review at the University of Nottingham and performed under home office licence PP5768261. Female mice BALB/c 19-22 g were housed in individually vented cages under a 12 h light cycle, food, and water ad libitum. Animals were prepared 1 hour prior to subcutaneous implantation of support by administration of Carprofen (2.5 mg/kg), anaesthetised with 2% isoflurane, their flanks shaved, and the skin cleaned with Hydrex surgical scrub. A small incision was made and UV sterilised 40mJ/cm^2^ 5-minute exposure per side in a Spectro linker XL-1000 test material implanted under surgical conditions beneath the PC muscle via a 9 g trocar needle and closed with GLUture skin glue (Abbott Laboratories). The mice were then allowed to recover and monitored daily. The implant samples used were non-commercial knitted polyethylene terephthalate (PET) meshes 7 × 2.4 mm^2^, provided by Medtronic, for R&D use only. For post-mortem experiments, animals were euthanised by cervical dislocation (schedule-1 procedure) at appropriate time points. For in-vivo DRS experiments, animals were sedated using Antisedan, after which the animals were euthanised by cervical dislocation (schedule-1 procedure), and the implant and surrounding tissue were isolated and fixed in 10% buffered formal saline and subjected to pathological analysis. For the post-mortem measurements, we used 2 animals per time point (Day 14, 21 and 28 post-implantation). Similarly, 2 animals per time point were also used for the *in-vivo* experiments (Day 7 and Day 28 post-implantation).

### 2.2. Histology

Tissue excised from the implantation sites were fixed and embedded into paraffin wax blocks, then cut into 7 µm parallel sections and mounted on APES (3-aminopropyl-ethoxysiliane) treated microscope slides. The sections were dried overnight and then deparaffinized in Xylene, rehydrated through graded alcohol series into distilled water.

Haematoxylin and eosin staining was carried out, based on the following protocol: Haematoxylin stain (4 min), wash in excess deionised water; Differentiate in 1% acid alcohol (1 dip), wash in tap water and blue in 1% lithium carbonate, 1 min, wash in tap water and counterstain in 1% eosin (2 min), wash in tap water and dehydrate through graded alcohols, equilibrate in Xylene for 10 min prior to mounting with cover glass. Bright field image tile mapping of tissue section was acquired on Zeiss Axioscan 7, 20× objective magnification.

Collagen fibre mapping was carried out using Picrosirius red (PR) staining kit (CliniSciences Cat 24901-500) as described in manufacturers protocol. Polarised image tile mapping of tissue section was acquired on Zeiss Axioscan 7, 20× objective magnification.

### 2.3. DRS instrument and data processing

The DRS instrument utilised a laser with a wavelength of 785 nm delivered through a Powell lens, which created a line-shaped laser spot of 10 × 1.5 mm^2^. To ensure the power density was equal to the skin MPE limit (3 mW/mm^2^), the total laser power was set to 45 mW. Dooley *et. al.* previously showed that a linear illumination provides the best trade-off between delivering high laser power, selection of spatial offsets for Raman detection, SORS probe design, while ensuring the laser power density on the tissue sample is compatible with the maximum permissible exposure [11]. Also, the simple linear shape for illumination allowed the use of a linear arrangement of fibre optics for collection of the Raman photons to match the length of the laser excitation line.

The Raman scattered light was collected by a custom-built fibre bundle consisting of 205 μm core diameter fibres (AMS Technologies). At the sample end, the fibres were set linearly in a 27 × 3 array imaged onto the surface of the sample using two lenses (focal lengths 50 mm focal and 30 mm, respectively). This arrangement provided a total collection area of 5.4 × 0.5 mm^2^, which is approximately 20-fold higher in the collection area than the previous DRS instrument [11]. At the spectrometer end, the optical fibres were arranged in a single line (81 fibres) to match the slit of the spectrometer (Princeton Instruments ISO320 with a Pixis2048 CCD). The schematic of the instrument is presented in Supplement 1, Figure S1.

For each measurement, the total integration time was 900 s (consisted of 9 individual measurements, each of 100 s exposure time), which is compatible with current relevant procedures for imaging small animals under anaesthetic [13]. Data pre-processing consisted of the following steps: 1) the CCD frames of the nine individual measurements were summed to obtain a single CCD image; 2) vertical binning of the summed CCD frame was used to obtain a single spectrum, combining the individual spectra from all 81 fibres; 3) a measured spectrum of polystyrene was used calibrate the wavenumber axis; 4) baseline removal was performed using an in-house asymmetric least squares algorithm; 5) spectra were normalised to the 1450 cm^−1^ peak (intensity equal to unity).

### 2.4. Modelling and optimisation of the DRS instrument

The computational models were based on NIRFASTer software, which was developed for modelling diffuse optical imaging and tomography [14,15]. The software is based on finite element modelling and has been used for the optimisation of diffuse Raman spectroscopy [11,12].

The schematic of the mouse model used in this study, including the skin layers and woven PET fibres implanted under the skin, is presented in Fig. 3(B). The finite element model was set up with a uniform mesh across the 3-D sample with 0.6 mm distance between nodes. In the area around the implant, the distance between nodes was reduced to 0.08 mm to increase the resolution of the results. The optimisation model mimicked the DRS instrument, including 35 × 3 excitation points arranged in a line (aspect ratio ∼ 10:1) and 441 detectors arranged in a 21 × 21 grid. Maps of Raman signal intensity at the skin surface (size 7 mm × 14 mm) were generated to determine the spatial offset that would generate the maximum signal to noise ratio (SNR) for collagen Raman signal. The metric chosen for calculating the signal to noise ratio (SNR) for the Raman signal from collagen (930 cm^−1^) was based on methods described previously [11]. The models were adapted to include reference Raman spectra of skin, muscle, adipose tissue, PET fibres and collagen (bovine Achilles tendon powder, Sigma-Aldrich product code C9879), all recorded with the excitation and detection point in the same position (0 mm offset). The Raman signal for collagen was calculated as the number of Raman photons in the 910–950 cm^−1^ band of the measured spectrum (after subtraction of a local linear baseline). The noise, which was dominated by photon shot noise, was calculated as the square-root of the total number of photons collected (including the background photons) in this wavenumber region and was estimated as the square-root of the total photon count. The SNR was calculated using Eq. (1) in Dooley *et. al.* (2023) [11].

## 3. Results

### 3.1. Histological evaluation of foreign body response to the woven PET fibres in the subcutaneous model

**Fig. 1.**
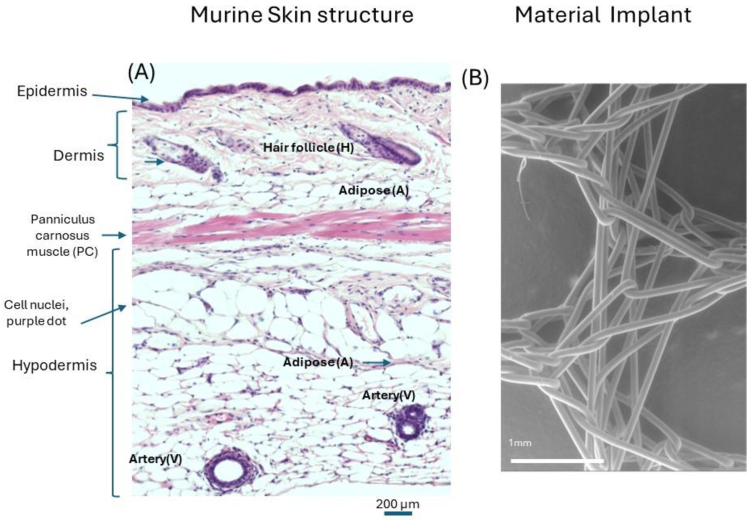
(A), Haematoxylin and eosin-stained histology showing normal mouse skin section **H**: hair follicle, **A**: adipose tissue **V**: arterial vessel, **PC**: panniculus carnosus muscle. Magnification 20× and scale bar 200 µm. (B) scanning electron microscopy (SEM) image of polyethylene terephthalate (PET) woven fibres. Magnification 50×, scale bar 1 mm.

Mouse and human skin is organised into three structural layers: the epidermis, dermis and hypodermis shown in Fig. 1(A). Immune cells, keratinocytes and fibroblasts cells reside within these layers amongst the vasculature, adipose and muscular layers. Using haematoxylin and eosin (H&E) stain, enables the observation of changes in cellular infiltration of cells using haematoxylin nuclei stain and the skin architectural structure can be demonstrated using the eosin stain.

To understand the ability of DRS to measure the molecular changes related to FBR *in-vivo*, FBR was induced by subcutaneous implantation of woven PET fibres as seen before implantation, according its anatomical location in the subcutaneous space (Fig. 1(B)).

**Fig. 2.**
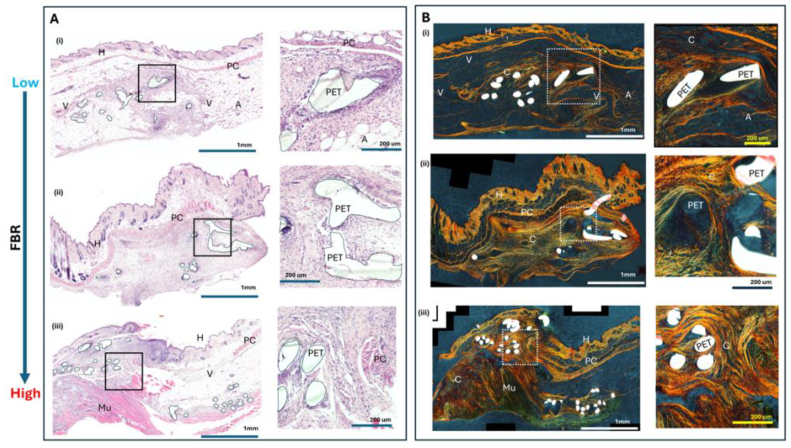
Typical examples of FBR responses in mouse dermal tissue after subcutaneous implantation of PET fibres. (A), Haematoxylin and eosin-stained histology showing mouse dermal response to PET fibres. **H**: hair follicle, **A**: adipose tissue **V**: Vien/arterial vessel, **Mu**: Latissimus Dorsi muscle **PC**: panniculus carnosus muscle. I/_*_ immune cell infiltration Magnification 20× and scale bar 1 mm, expanded panel scale bar 200 µm. (B) Polarised light acquisition of Picrosirius red staining of collagen fibres in parallel sections to haematoxylin and eosin stain, showing birefringence detection of collagen fibres and white PET fibres. Magnification 20× and scale bar 1 mm, expanded panel scale bar 200 µm. PET fibre implantation site highlighted by black line and labelled **PET**.

Before performing the DRS measurement, we tested and validated the level of FBR induced by the PET fibres (Fig. 2). PET fibres implanted under the panniculus carnosus muscle have been reported to induce an obvious FBR as indicated by increased staining of collagen fibres with Picrosirius red (PR), an anionic dye which reacts with basic amino acids between collagen fibres in the tissue [16]. PR staining in Fig. 2(B) showed that there was a heterogeneity in response depending on PET fibres proximity to structures in the dermis. Proximity to both the latissimus dorsi muscle and panniculus carnosus muscle produced a higher level of collagen fibres, whilst when the PET fibres were next to the adipose tissue pocket a reduced levels of PR was seen indicating a reduced level of collagen fibres.

For the sample presented in Fig. 2 A(i) and B(i), the PET fibres are encapsulated in a dermal adipose pocket, which has not produced a defined FBR collagen capsule in the PR image.

The PET fibres implanted in the dermal structures which are close to the latissimus dorsi muscle, as in Fig. 2 A(ii) and A(iii), induced a stronger FBR response and led to increased collagen fibres as shown by the increased birefringence Picrosirius Red positive tissue (Fig. 2 B(ii) and B(iii)). Surrounding each encapsulated PET fibre there is an infiltrated cellular layer between the fibre and the collagen fibre response, seen in Fig. 2(B)(i) zoom image inserts.

The PET fibres implanted in the dermal structures which are both close to the latissimus dorsi muscle and an adipose pocket produce a heterogeneous response. Overall, higher FBR response (Fig. 2 A(iii) and B(iii)) is observed in cases associated with PET fibres implanted in the dermal structures close to the latissimus dorsi muscle and panniculus carnosus muscle, compared with the FBR response in the subcutaneous adipose tissue, which shows a reduction in collagen fibre formation within the same tissue site.

### 3.2. Raman spectroscopy of skin tissue and implant materials

**Fig. 3.**
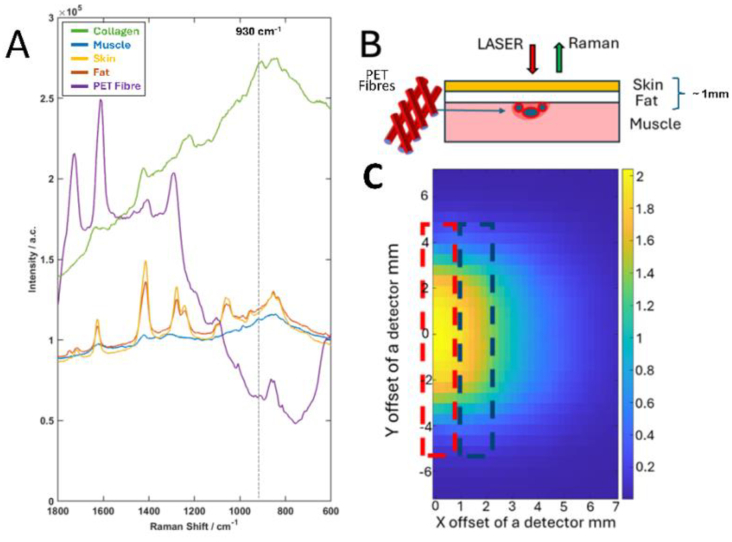
(A) Reference Raman spectra of collagen, skin, muscle, adipose tissue, and woven PET fibres. The guideline indicates the main Raman band assigned to collagen at 930 cm^−1^. (B) Schematic and numerical modelling results for the woven PET fibres implanted under the skin of a mouse model. (C) The surface map of the 3D model showing the SNR for the 930 cm^−1^ Raman band (assigned to collagen) calculated at each individual detector point when using a single-line laser excitation (indicated by the red dotted rectangle). The blue dotted rectangle indicates the collection points for the Raman photons (in the experimental set-up made up of 3 × 27 fibres focused on the surface).

The fibrous tissue that incapsulates a foreign body is generally formed by accumulation of collagen. The reference Raman spectra of the PET fibres, collagen purchased commercially, and other tissue components (muscle, skin, adipose tissue) isolated from mice are presented in Fig. 3(A).

The Raman bands of the PET fibres were detected at 1266 cm^−1^ CH symmetric bending, 1413 cm^−1^ CH asymmetric bending, 1616 cm^−1^ C-C/C = C benzene ring mode, and 1729cm^−1^ C = O stretching (Fig. 3(A)). These bands correspond to polyethylene terephthalate (PET), the primary material used in the surgical mesh, as reported in the literature [17]. The Raman band at 1450 cm^−1^ corresponding to C-H_2_ scissoring is one of the representative peaks that can be found in biological Raman spectra, correlating to the protein and lipid compositions in the sample [18], this band has widely been used for normalisation due to its consistency and this peak is not present in the Raman spectrum of PET, i.e. bio-material-significant only in this case [19]. The Raman spectrum of collagen shows its characteristic bands at 850 and 930 cm^−1^, arisen from the proline and hydroxyproline in collagen. However, Raman signal of collagen can also be found in the typical Amide III and Amide I bands at 1200-1350 cm^−1^ and 1660 cm^−1^ which are the peaks consisting combinations of multiple biomolecular compositions [20]. The Amide I and Amide III bands are also present in the Raman spectra of skin and muscle. The Raman spectra of skin and subcutaneous fat also contain Raman bands assigned to molecular vibrations of lipid molecules, such as the C = C stretching at 1670 cm^−1^, C-H deformations at 1250-1350 cm^−1^, skeletal C-C stretches at 1030-1150 cm^−1^, and CH deformations at 890 cm^−1^ [18]. Although most of the Raman bands of collagen overlap with the Raman bands of skin components and implant materials, the 930 cm^−1^ band has the least overlapping region in a Raman spectrum and hence was chosen to use in this study to express the abundance of collagen in tissue, as an indicator of FBR.

Figure 3(C) shows numerical modelling results for the calculated signal-to-noise ratio (SNR) corresponding to the 930 cm^−1^ collagen Raman band, simulating the condition of using woven PET fibres as the implant. The results indicate the maximum SNR ≅ 2 can be achieved at each detection point, corresponding to a spatial offset of ∼1.4 mm. Thus, a spatial offset between the laser excitation and detection position was set at 1.5 mm for all measurements.

### 3.3. Post-mortem DRS measurements of FBR induced by PET fibres

**Fig. 4.**
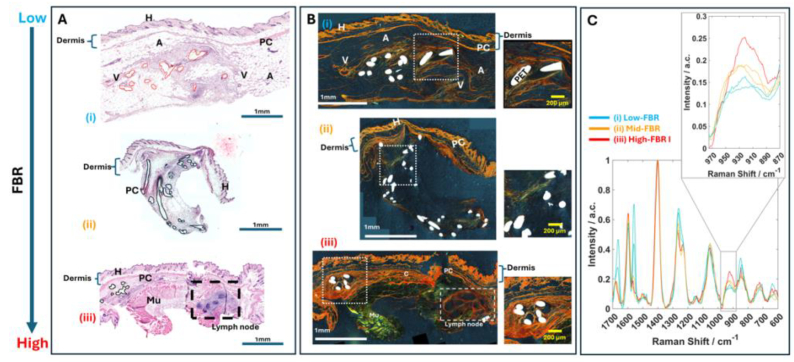
Comparison of *in-situ* DRS spectra measured post-mortem from mice at the implantation site of PET fibres. Histology sections of the skin tissue at the implantation site (after tissue excision) are presented for reference. (A) Histology images for typical cases in order of increasing FBR Haematoxylin and eosin-stained histology **H**: hair follicle, **A**: adipose tissue, **Mu**: Latissimus Dorsi muscle **V**: arterial vessel, **PC**: panniculus carnosus muscle. (B) Polarised light acquisition of Picrosirius red stain on sections of the skin tissue at the implantation site. Magnification 20× and scale bar 1 mm. C) *in-situ* DRS spectra of all animals measured post-mortem.

The DRS spectra measured *in-situ* at the site of implantation of PET fibres are shown in Fig. 4. The DRS measurements were recorded post-mortem to permit establishment of optimal parameters for DRS spectra acquisition in live animals. Given the heterogeneity of FBR presented in section 3.1, Raman spectra were measured at 14-, 21- and 28 days post implantation, and the level of FBR (from low to high) was established after evaluation of the histology images (H&E and polarised light PR staining).

The intensity of the 930 cm^−1^ Raman band was found to increase with the level of the FBR, according to the histology results in Fig. 4(A) and 4(B). The lowest level of FBR was observed when the PET fibres were implanted in subcutaneous adipose pockets (Fig. 4(A)(i)), and a low amount of collagen was observed in Fig. 4(B)(i). The DRS spectra corresponding to these samples indicated the lowest intensity of the 930 cm^−1^ Raman band corresponding to collagen (Fig. 4(C)(i)).

Figure 4(A)(ii) shows a case when the PET fibers was implanted directly under the skin, leading to a large area of surrounding PS-stained fibrotic tissue around the implant (Fig. 4(B)(ii)). Stronger PS-stained fibrotic tissue was observed when the implant was in an area close to multiple lymph nodes (long dash box in Fig. 4(A)(iii) and 4B(iii)).

### 3.4. Live animal acquisition of in-vivo DRS measurements of FBR induced by PET fibres

**Fig. 5.**
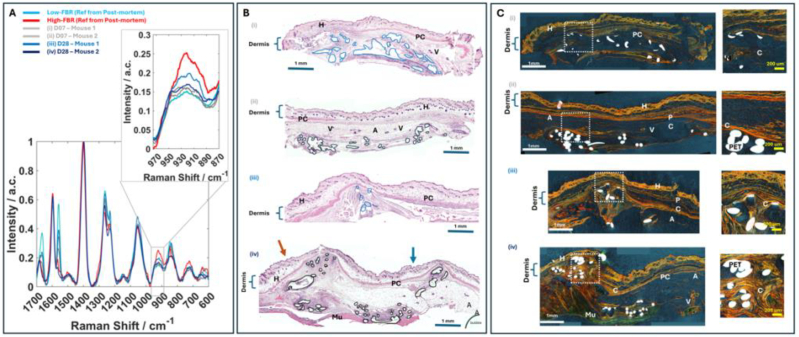
(A) *in-vivo* DRS spectra measured at 7- and 28-days post-implantation (grey and black lines). Mean DRS spectra from post-mortem experiments (low and high FBR) are included for comparison (blue and red lines). (B) Haematoxylin and eosin-stained histology images for typical cases in order of increasing FBR **H**: hair follicle, **A**: adipose tissue, **Mu**: Latissimus Dorsi muscle **V**: arterial vessel, **PC**: panniculus carnosus muscle. C) Polarised light acquisitions of Picrosirius red stained parallel sections of skin tissue at the implantation site. Magnification 20× and scale bar 1 mm.

*In-vivo* DRS measurements were carried out on mice after subcutaneous implantation of woven PET fibres (Fig. 5). Measurements were recorded at the implantation site, from individual mice at days 7 and 28 post-implantation, intended to detect spectra changes related to low (7 day) and high (28 day) FBR response *in-vivo*.

For all live animal measurements, the *in-vivo* recorded DRS have a similar pattern of bands as the spectra from the post-mortem measurements, as shown in Fig. 5(A). The DRS spectra measured from mice at Day 7 implantation (grey spectra) show similar level of collagen signal at the selected 930 cm^−1^ band as the low FBR post-mortem mean spectrum. This was cross validated with the histology results presented in Fig. 5(B) and Fig. 5(C), which confirm a low level of collagen fibres.

One of the *in-vivo* Raman spectra from the live-animals at Day 28 post-implantation (Fig. 5(A)(iii)) gives higher intensity at the selected 930 cm^−1^ collagen peak, suggesting higher collagen concentration in the subcutaneous tissue. This result is confirmed by the corresponding histology results in Fig. 5(B) (iii) & 5C (iii), showing a stronger FBR and higher level of collagen fibres. In addition to the bio-molecular signals acquired, intense Raman peaks at 1616 cm^−1^ and 1729cm^−1^ were also observed, suggesting that the PET filers were closer to the skin surface. The histology results confirm this observation further as the images show that the muscle below the PET fibres adhered directly to the skin, and hence pushing the implant closer to the skin surface. Consistent with this, the DRS spectrum in Fig. 5(A) (iv) shows a lower 930 cm^−1^ collagen band (at a level expected for a low FBR level) and relatively low intensity bands at 1616 and 1729cm^−1^ assigned to the PET. The histology images corresponding to this sample (Fig. 5(B) (iv) and 5C (iv)) show that significant portion of FBR surrounding the PET fibres close to the skin surface on the left-hand side of the image (red arrow). However, on the right-hand side part of the histology image shows a similar amount of woven PET fibres surrounded by a high level of FBR deeper in the subcutaneous tissue, below a large fat pad (blue arrow). One explanation of the low intensity of the 930 cm^−1^ band in the DRS spectrum could be that the DRS measurement was recorded from the skin area corresponding to the right-hand side PET fibres (blue arrow), approximately twice as deep under the skin compared to the left-hand side (red arrow), and the Raman photons corresponding to collagen and the PET fibres were attenuated more before escaping to the surface to reach the detector. These results indicate that higher accuracy in the lateral positioning of the DRS probe is required in order to accurately detect and quantify collagen signals from the subcutaneous tissue affected by the FBR.

## 4. Discussion

This paper investigated for the first time the ability of using DRS to measure *in-vivo* biomolecular changes in skin and subcutaneous tissue related to FBR after subcutaneous implantation of biomaterials. As the first step, post-mortem mice were used to determine the optimal acquisition parameters and the feasibility to detect different levels of FBR *in-situ*. Tissue from the same DRS measurement sites were excised after Raman acquisitions for histological investigations. Overall, the results show a strong correlation between the FBR and the collagen content, which can be indicated with the intensity of the selected collagen Raman band at 930 cm^−1^. This 930 cm^−1^ band acts as a good FBR level indicator as it does not overlap with other Raman bands assigned to skin tissue or the implanted PET fibres. Although elastin, is a key component of the skin, its concentration is significantly lower than collagen (usually, one tenth), being perhaps below the detection limit of the instrument.

After optimisation, DRS measurements were performed on live animals to demonstrate the feasibility of recording DRS *in-vivo* with live mice under anaesthetic. The DRS spectra acquisition in live-animal results were consistent with the post-mortem results and cross-validated by the histology results, which confirmed the correlation between the intensity of the selected 930 cm^−1^ Raman peak and FBR levels. However, the live-animal results in this study also show that a reliable quantitative analysis of the FBR based on the DRS spectra is challenging. Uncertainties related to the depth of the implant can lead to variations in the intensity of the Raman bands. Thus, methods to account for both the uncontrollable experimental and biological variations that can affect the intensity of the 930 cm^−1^ collagen band are required before collagen or FBR quantification can be developed. The 3D location of the foreign body is very important, as it affects the path travelled by the photons reaching the detector, which affects the attenuation of the Raman bands. Although the location of the implant was marked on the animal skin, the implant can move inside the subcutaneous tissue while the mice grow. These uncertainties could be reduced by coupling the DRS instrument with a second imaging probe, such as ultrasound or optical coherence tomography, to allow accurate location of the sampling area around the implant. A second imaging probe could also provide extra information on the skin thickness above the implant, which could be used to normalise the DRS results. The advantages of such dual-modality measurement would allow time-course measurements able to provide data with sufficient statistical significance regarding tissue morphology and biomolecular composition that can be reliably related to FBR.

## 5. Conclusions

This study demonstrates the feasibility of using diffuse Raman spectroscopy (DRS) to detect *in-vivo* spatial molecular changes in mouse skin caused by foreign body response (FBR) after subcutaneous implantation of surgical mesh. The intensity of the 930 cm^−1^ Raman band corresponding to collagen was found to have a positive correlation with the level of FBR observed by histology. Nevertheless, the study also highlights the challenges regarding the quantification of FBR based on DRS due to the uncontrollable experimental and biological factors. However, such difficulties could be overcome by coupling the DRS system to another non-invasive imaging technique that could provide information regarding the location of the implant and, possibly, dual imaging information of collagen and synthetic polymers such as PET which should be the case with OCT. Such integration would allow *in-vivo* quantification of FBR in a non-invasive way, enabling longitudinal spatial distribution measurements on the same animal, rather than sacrificing animals at specific time points for standard histology assessment. In longer term this approach could also be applied to a range of animal models, include applications beyond FBR, and potentially to humans for diagnosis or treatment monitoring, such as regeneration of connective tissue, wound healing, inflammatory diseases, etc.

## Supplemental information

Supplement 1 Supplemental Document https://doi.org/10.6084/m9.figshare.29918444

## Data Availability

Data underlying the results presented in this paper are freely available at the University of Nottingham data repository [21].

## Supplemental document

See Supplement 1 for supporting content.

## References

[1] Morais J. M. Papadimitrakopoulos F. Burgess D. J., “Biomaterials/tissue interactions: Possible solutions to overcome foreign body response,” AAPS J. 12(2), 188–196 (2010). 10.1208/s12248-010-9175-3 20143194 PMC2844517

[2] Franzen L. Windbergs M., “Applications of Raman spectroscopy in skin research — From skin physiology and diagnosis up to risk assessment and dermal drug delivery,” Adv. Drug Delivery Rev. 89, 91–104 (2015). 10.1016/j.addr.2015.04.002 25868454

[3] Kong K. Rowlands C. J. Varma S. et al., “Diagnosis of tumors during tissue-conserving surgery with integrated autofluorescence and Raman scattering microscopy,” Proc. Natl. Acad. Sci. U. S. A. 110(38), 15189–15194 (2013). 10.1073/pnas.1311289110 24003124 PMC3780864

[4] Caspers P. J. Lucassen G. W. Carter E. A. et al., “In Vivo Confocal Raman Microspectroscopy of the Skin: Noninvasive Determination of Molecular Concentration Profiles,” J. Invest. Dermatol. 116(3), 434–442 (2001). 10.1046/j.1523-1747.2001.01258.x 11231318

[5] Su J. W. Wang Q. Tian Y. et al., “Depth-sensitive Raman spectroscopy for skin wound evaluation in rodents,” Biomed. Opt. Express 10(12), 6114 (2019). 10.1364/BOE.10.006114 31853389 PMC6913421

[6] Matousek P. Morris M. D. Goodship A. E. et al., “Subsurface Probing in Diffusely Scattering Media Using Spatially Offset Raman Spectroscopy,” Appl. Spectrosc. 59(4), 393–400 (2005). 10.1366/0003702053641450 15901323

[7] Matousek P. Draper E. R. C. Goodship A. E. et al., “Noninvasive Raman spectroscopy of human tissue in vivo,” Appl. Spectrosc. 60(7), 758–763 (2006). 10.1366/000370206777886955 16854263

[8] Bergholt M. S. Albro M. B. Stevens M. M., “Online quantitative monitoring of live cell engineered cartilage growth using diffuse fiber-optic Raman spectroscopy,” Biomaterials 140, 128–137 (2017). 10.1016/j.biomaterials.2017.06.015 28649013 PMC5504667

[9] Liao Z. Sinjab F. Nommeots-Nomm A. et al., “Feasibility of Spatially Offset Raman Spectroscopy for in Vitro and in Vivo Monitoring Mineralization of Bone Tissue Engineering Scaffolds,” Anal. Chem. 89(1), 847–853 (2017). 10.1021/acs.analchem.6b03785 27983789

[10] Dooley M. McLaren J. Rose F. R. A. J. et al., “Investigating the feasibility of spatially offset Raman spectroscopy for in-vivo monitoring of bone healing in rat calvarial defect models,” J. Biophotonics 13(10), e202000190 (2020). 10.1002/jbio.202000190 32658374

[11] Dooley M. Luckett J. Alexander M. R. et al., “Optimization of diffuse Raman spectroscopy for in-vivo quantification of foreign body response in a small animal model,” Biomed. Opt. Express 14(12), 6592 (2023). 10.1364/BOE.512118 38420302 PMC10898571

[12] Dooley M. Paterson T. Dexter L. et al., “Model-Based Optimization of Laser Excitation and Detection Improves Spectral Contrast in Noninvasive Diffuse Raman Spectroscopy,” Appl. Spectrosc. 76(7), 801–811 (2022). 10.1177/00037028211072900 35081779

[13] Mills B. Awais R. O. Luckett J. et al., “[18F]FDG-6-P as a novel in vivo tool for imaging staphylococcal infections,” EJNMMI Res. 5(1), 13 (2015). 10.1186/s13550-015-0095-1 25853019 PMC4385282

[14] Jermyn M. Ghadyani H. R. Mastanduno M. A. et al., “Fast segmentation and high-quality three-dimensional volume mesh creation from medical images for diffuse optical tomography,” J. Biomed. Opt. 18(8), 086007 (2013). 10.1117/1.JBO.18.8.086007 23942632 PMC3739873

[15] Dehghani H. Eames M. E. Yalavarthy P. K. et al., “Near infrared optical tomography using NIRFAST: Algorithm for numerical model and image reconstruction,” Commun. Numer. Meth. Engng. 25(6), 711–732 (2009). 10.1002/cnm.1162 PMC2826796 20182646

[16] Berger R. Filho J. M. R. Malafaia O. et al., “Histological evaluation of capsules formed by texturized silicone implants with and without polyester mesh coverage (Parietex®). A study on female rats,” Acta. Cir. Bras. 36(5), e360505 (2021). 10.1590/acb360505 34133505 PMC8205442

[17] Rebollar E. Pé rez S. Herná ndez M. et al., “Physicochemical modifications accompanying UV laser induced surface structures on poly(ethylene terephthalate) and their effect on adhesion of mesenchymal cells †,” Phys. Chem. Chem. Phys 16(33), 17551 (2014). 10.1039/C4CP02434F 25025655

[18] Movasaghi Z. Rehman S. Rehman I. U., “Raman spectroscopy of biological tissues,” Appl. Spectrosc. Rev. 42(5), 493–541 (2007). 10.1080/05704920701551530

[19] Afseth N. K. Segtnan V. H. Wold J. P., “Raman spectra of biological samples: A study of preprocessing methods,” Appl. Spectrosc. 60(12), 1358–1367 (2006). 10.1366/000370206779321454 17217584

[20] Shipp D. W. Sinjab F. Notingher I., “Raman spectroscopy: techniques and applications in the life sciences,” Adv. Opt. Photonics 9(2), 315–428 (2017). 10.1364/AOP.9.000315

[21] Notingher I., “Feasibility of diffuse Raman spectroscopy to detect in-vivo molecular changes in the tissue induced by subcutaneous implants: data University of Nottingham, 2025, 10.17639/nott.7558 PMC12684047 41368098

