# Supplementary material for: Feasibility of diffuse Raman spectroscopy to detect in-vivo molecular changes in the tissue induced by subcutaneous implants
Source: Biomed Opt Express. 2025 Aug 22;16(9):3759–69. doi: 10.1364/BOE.567960 (PMC12684047; doi:10.1364/BOE.567960)
Supplement: Supplementary file 1 [file boe-16-9-3759-s001.pdf]

Supplemental Document

Biomedical Optics EXPRESS

Feasibility of diffuse Raman spectroscopy to detect in-vivo molecular changes in the tissue induced by subcutaneous implants: supplement

MAX DOOLEY,[1] JENI LUCKETT,[2] NGA TSING TANG,[1] MORGAN R. ALEXANDER,[3] PAVEL MATOUSEK,[4] HAMID DEHGHANI,[5] YVES BAYON,[6] AMIR M. GHAEMMAGHAMI,[2] AND IOAN NOTINGHER[1,*]

[1] *School of Physics and Astronomy, University of Nottingham, University Park, Nottingham NG7 2RD, UK*
[2] *School of Life Sciences, University of Nottingham, University Park, Nottingham NG7 2RD, UK*
[3] *School of Pharmacy, University of Nottingham, University Park, Nottingham NG7 2RD, UK*
[4] *Central Laser Facility, Research Complex at Harwell, STFC Rutherford Appleton Laboratory, UK Research and Innovation (UKRI), Harwell Oxford OX11 0QX, UK*
[5] *School of Computer Science, University of Birmingham, Birmingham B15 2TT, UK*
[6] *Sofradim Production – 116 Avenue du Formans – 01600 Trévoux, France*
[*] *ioan.notingher@nottingham.ac.uk*

This supplement published with Optica Publishing Group on 22 August 2025 by The Authors under the terms of the Creative Commons Attribution 4.0 License in the format provided by the authors and unedited. Further distribution of this work must maintain attribution to the author(s) and the published article's title, journal citation, and DOI.

Supplement DOI: https://doi.org/10.6084/m9.figshare.29918444

Parent Article DOI: https://doi.org/10.1364/BOE.567960

Feasibility of diffuse Raman spectroscopy to detect in-vivo molecular changes in the tissue induced by subcutaneous implants

Supplementary Document

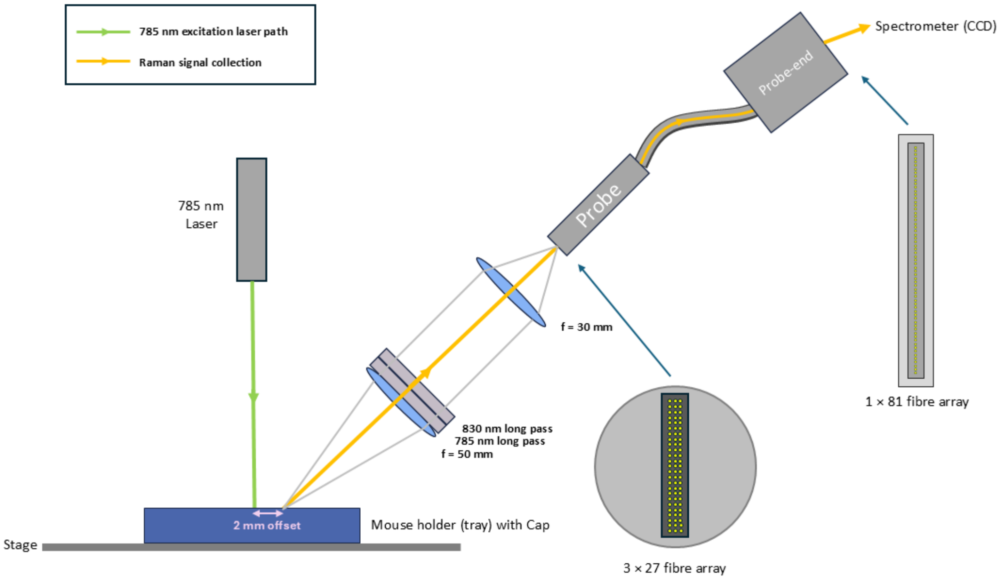

Figure S1. Schematic of the DRS system used for post-mortem and *in-vivo* work, with fibre-probes arrays layout stated.
